# Anti-Adipogenic Activity of *Rhaponticum carthamoides* and Its Secondary Metabolites

**DOI:** 10.3390/nu15133061

**Published:** 2023-07-07

**Authors:** Velislava Todorova, Martina S. Savova, Stanislava Ivanova, Kalin Ivanov, Milen I. Georgiev

**Affiliations:** 1 Department of Pharmacognosy and Pharmaceutical Chemistry, Faculty of Pharmacy, Medical University-Plovdiv, 4002 Plovdiv, Bulgaria; velislava.todorova@mu-plovdiv.bg (V.T.); kalin.ivanov@mu-plovdiv.bg (K.I.); 2 Laboratory of Metabolomics, Institute of Microbiology, Bulgarian Academy of Sciences, 4000 Plovdiv, Bulgaria; m.sav@abv.bg (M.S.S.); milengeorgiev@gbg.bg (M.I.G.)

**Keywords:** *Rhaponticum carthamoides* (Willd.) Iljin., 20-hydroxyecdysone, ponasterone A, turkesterone, high-performance thin layer chromatography (HPTLC), obesity, adipocytes, adipogenesis, adipolysis

## Abstract

Besides their common use as an adaptogen, *Rhaponticum carthamoides* (Willd.) Iljin. rhizome and its root extract (RCE) are also reported to beneficially affect lipid metabolism. The main characteristic secondary metabolites of RCE are phytoecdysteroids. In order to determine an RCE’s phytoecdysteroid profile, a novel, sensitive, and robust high-performance thin-layer chromatography (HPTLC) method was developed and validated. Moreover, a comparative analysis was conducted to investigate the effects of RCE and its secondary metabolites on adipogenesis and adipolysis. The evaluation of the anti-adipogenic and lipolytic effects was performed using human Simpson–Golabi–Behmel syndrome cells, where lipid staining and measurement of released glycerol and free fatty acids were employed. The HPTLC method confirmed the presence of 20-hydroxyecdysone (20E), ponasterone A (PA), and turkesterone (TU) in RCE. The observed results revealed that RCE, 20E, and TU significantly reduced lipid accumulation in human adipocytes, demonstrating their anti-adipogenic activity. Moreover, RCE and 20E were found to effectively stimulate basal lipolysis. However, no significant effects were observed with PA and TU applications. Based on our findings, RCE and 20E affect both lipogenesis and lipolysis, while TU only restrains adipogenesis. These results are fundamental for further investigations.

## 1. Introduction

Obesity and the state of being overweight are among the continuously growing public health concerns [1,2,3,4]. According to the World Health Organization (WHO), currently, almost 2 billion adults are considered overweight and 650 million of them are considered obese [5]. Common comorbidities attributed to obesity and the state of being overweight are hypertension, dyslipidemia, type 2 diabetes, sleep apnea, osteoarthritis, and cancer [2,6,7,8]. Current strategies for excess adiposity management include lifestyle modifications, pharmacotherapy, and bariatric surgery in the most severe cases [8,9,10]. However, the number of approved and safe drugs for the reduction of body weight is quite limited—orlistat, phentermine, topiramate, and semaglutide [11,12,13]. At present, plenty of studies are focused on the research of novel molecules with anti-obesity activity [14]. Many of these potential therapeutic molecules are plant secondary metabolites [15,16,17]. Numerous plants are reported to possess anti-obesity potential such as *Eleutherococcus senticosus, Bassia scoparia*, *Platycodon grandiflorum*, *Gypsophila oldhamiana*, *Momordica charantia*, *Rosmarinus officinalis*, *Citrus limon*, *Taraxacum officinale*, and *Ziziphus jujuba* [1,18,19]. The anti-obesity activity of plant species is related to their phytochemical content, especially: saponins (platycodin A, platycodin C, deapioplatycodin D, momordin Ic, escin Ia, escin IIa, escin Ib, etc.), polyphenols (caffeic acid, chlorogenic acid, resveratrol, curcumin, kaempferol, quercetin, cyanidin, naringenin, etc.), terpenoids (lycopene, lutein, and carotene), organosulfurs (allicin and alliin), and phytosterols (protodisoscin and diosgenin) [18,20]. These natural compounds may exert their anti-obesity effect through more than one of the following mechanisms: inhibition of pancreatic lipases, stimulation of lipolysis, inhibition of differentiation of preadipocytes, stimulation of adipose tissue browning, and induction apoptosis of existing hypertrophied adipocytes [18,20].

**Figure 1:**
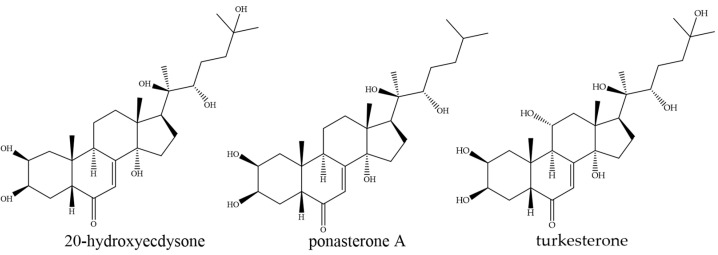
Chemical structures of the phytoecdysteroids–20-hydroxyecdysone, ponasterone A and turkesterone.

Although many bioactive compounds of plant origin possess anti-obesity potential, the specific role of phytoecdysteroids (PDs) remains incompletely understood. Noratto et al. reported that the intake of quinoa (a phytoecdysteroid-rich plant) is associated with positive effects on obesity in mice [21]. However, further research is needed to reveal the potential of PDs and phytoecdysteroid-containing plants on obesity. Among the phytoecdysteroid-rich plants is the endemic perennial plant *Rhaponticum carthamoides* (Willd.) Iljin. from the Asteraceae family, commonly known as maral root or Russian leuzea [22,23]. In traditional medicine, it has been used to improve physical strength [22]. In 1969, leuzea was systemized as one of the plant adaptogens by Brekman and Dardimov [22]. In recent decades, extracts from its rhizomes and roots have been used for physical weakness, to promote muscle growth, to treat impotency, etc. [22]. Various medicinal preparations from *R. carthamoides* rhizomes and roots have been reported to possess not only adaptogenic effects but also a broad spectrum of biological effects, such as antioxidant, immunomodulatory, anticancerogenic, antimicrobial, antiparasitic, and repellent activities [22,23,24]. The main isolated chemical classes from *R. carthamoides* rhizomes and roots are not only PDs but also phenolics (flavonoids and phenolic acids) [22]. Previous phytochemical reports regarding ecdysteroids of *R. carthamoides* from the underground parts of the plants revealed the isolation of 20-hydroxyecdysone, also known as ecdysterone (20E), ponasterone A (PA), and turkesterone (TU) [22,25,26]. Ecdysteroids are a group of polyhydroxylated sterols, structurally similar to androgens (Figure 1) [24,27,28].

PDs possess a wide range of pharmacological properties, including antidiabetic and hepatoprotective properties [24,29]. Previous studies reported that 20E possesses anabolic, neuroprotective, and antitumor effects, restores renal dysfunction, and decreases triglycerides [28,29,30,31,32,33,34]. It has the potential to prevent adiposity, dyslipidemia, and hyperglycemia [35,36], while PA possesses anabolic activity [29]. Turkesterone has been associated with anabolic, antidiabetic, and hypoazotemic effects, as well as the ability to decrease cholesterol levels and restore renal function, according to previous investigations [29].

The current study aimed to evaluate the phytochemical profile of *R. carthamoides* rhizomes and roots extract (RCE) via an innovative high-performance thin layer chromatography (HPTLC) method. Moreover, the PDs in the extract—20E, PA, and TU—were quantified. Following the chemical analysis, the effect of RCE as well as its secondary metabolites on adipogenesis and adipolysis were investigated in an in vitro obesity model of human adipocytes.

## 2. Materials and Methods

### 2.1. Chemicals and Reagents

The reference standard of PA (molecular weight: 464.6 g·mol^−1^; purity: HPLC ≥ 95%, #16,386) was purchased from Cayman Chemical, Ann Arbor, MI, USA. The reference standards of 20E (molecular weight 480.64 g·mol^−1^; purity: HPLC ≥ 95%, #89,651) and TU (molecular weight: 496.6 g·mol^−1^; purity: HPLC ≥ 95%, #85,781) were obtained from PhytoLab GmbH & Co. KG, Vestenbergsgreuth, Germany. Analytical grade dimethyl sulfoxide (DMSO), isopropanol, acetonitrile, methanol, cell culture medium Dulbecco’s modified Eagle’s medium/Nutrient F-12 Ham, Oil red O (ORO; 0.5% solution in isopropanol), fetal bovine serum, penicillin/streptomycin 10,000 IU/10 mg·mL^−1^, d-biotin (purity > 99%), d-pantothenic acid (purity > 99%), human apo-transferrin (purity > 98%), rosiglitazone (purity: HPLC > 98%), human insulin, 3-isobutyl-1-methylxantine (purity: HPLC > 99%), dexamethasone (purity: HPLC > 98%), triiodothyronine (purity > 95%), cortisol (purity > 95%), and isoproterenol hydrochloride (purity: HPLC > 98%) were obtained from Merck KGaA (Darmstadt, Germany).

### 2.2. Plant Material and Extraction

The dried *R. carthamoides* rhizomes and roots were purchased from Russia. The plant material was characterized according to the Russian Pharmacopoeia by the Department of Pharmacognosy and pharmaceutical chemistry, Medical University of Plovdiv. The plant material was further frozen, freeze-dried, and ground before ultrasound-assisted extraction with 50% aqueous methanol at 20 °C for 30 min. The obtained RCE was filtered and concentrated via a rotary vacuum evaporator at 40 °C, further freeze-dried, and stored at −20 °C before use.

### 2.3. HPTLC Analyses

#### 2.3.1. Sample Preparation

The stock solutions for HPTLC analysis of 20E, PA, T, and the extract were prepared in acetonitrile in concentration 1 mg·mL^−1^. Ultrasound was used for better dissolution. The prepared stock solutions were stored before use in brown vials, protected from light at 4 °C.

#### 2.3.2. Instrumentation

The method was developed using a CAMAG HPTLC system (CAMAG, Muttenz, Switzerland) in the following configuration: CAMAG Limomat 5, a software-controlled applicator of CAMAG, Muttenz, Switzerland); CAMAG Automatic Developing Chamber 2 (CAMAG, Muttenz, Switzerland), and CAMAG TLC Visualizer 2 (CAMAG, Muttenz, Switzerland). The software used was “VisionCATS” (version 3, CAMAG, Muttenz, Switzerland). An ultrasonic bath (Bandelin, Berlin, Germany) was used for better dissolution of the standard solutions.

#### 2.3.3. Method Development

The analyses were carried out using silica gel 60 F254 glass TLC plates of 10 × 20 cm size and with 200 μm layer thickness (E. Merck KGaA, Darmstadt, Germany). The mobile phase comprised methanol: acetonitrile at a ratio of 10:90 (*v*/*v*). The volume of the mobile phase was 10 mL. The application type was a band, and the front was 70 mm. The time for development was 10 min, followed by drying for 5 min. Detection was performed at 254 nm using CAMAG TLC Visualizer 2.

#### 2.3.4. Method Validation

The developed method was validated according to the International Council for Harmonization of Technical Requirements for Pharmaceuticals for Human Use (ICH) with the following validation parameters: linearity, range, precision, accuracy, and limits of detection (LD) and quantification (LQ) [37].

### 2.4. Cell Culture and Treatment

Human Simpson–Golabi–Behmel syndrome (SGBS) preadipocytes were kindly provided by Professor Martin Wabitsch (University of Ulm, Germany). The cells were cultured under optimal conditions [38,39]. The differentiation of near-confluent preadipocytes was carried out with the presence of RCE (5–50 μg·mL^−1^), 20E (5–50 μM), PA (5–50 μM), TU (5–50 μM), or 0.02% DMSO as a vehicle. These concentrations were selected based on cell viability evaluation with the 3-(4,5-dimethylthiazol-2-yl)-2,5-diphenyltetrazolium bromide (MTT) assay. The experimental treatments were applied upon differentiation and on the fourth and eighth days with every culture media replacement process. Sample collection and subsequent analyses were performed 24 h after the last treatment. Each assay was performed at least in three independent experiments.

### 2.5. Cell Viability Assay

The preadipocytes were seeded in 96-well plates, grown to near confluence, and incubated for 48 h with increasing concentrations from 0.1 to 100 μM for the pure compounds and 0.1 to 100 μg·mL^−1^ for the extract or 0.02% DMSO as a vehicle. The results are presented as the percentage of cell viability compared to the vehicle as the mean ± SEM and are representative of three independent experiments.

### 2.6. Lipid Staining

The procedure was performed as previously described [40]. Briefly, on day 9 of differentiation, the SGBS adipocytes were fixed with formalin and subsequently stained with freshly prepared ORO solution. Then, the representative microphotographs were taken using an Oxion Inverso OX.2053-PLPH inverted microscope, equipped with a DC.10,000-Pro CMEX camera (Euromex, Arnhem, The Netherlands). For the quantification of accumulated lipids for each group, the absorbance of the extracted lipid dye at 495 nm was measured using an Anthos Zenyth 340 multiplate reader from Biochrom Ltd. (Cambridge, United Kingdom). The results were represented as the percentage of accumulated lipids compared to the vehicle-treated group.

### 2.7. Analysis of Basal and Stimulated Lipolysis

The effect of increasing concentrations of RCE, 20E, PA, and TU, was evaluated through quantification of released glycerol and free fatty acids (FFAs) in the culture media, as products of lipid hydrolysis. Along with the last treatment, on day 8 of adipogenic differentiation, lipolysis was stimulated with 10 μM isoproterenol for 24 h [41]; then, culture media samples were collected from the tested treatments subjected to both basal and isoproterenol-stimulated conditions. Glycerol and FFA concentrations were determined using a glycerol assay kit (#MAK117) and a free fatty acid assay kit (#MAK044) from Merck KGaA according to the manufacturer’s instructions.

### 2.8. Statistical Analysis

The resulting data were expressed as the mean ± SEM and statistical significance between groups was determined by one-way ANOVA, followed by Tukey’s post hoc test, using SigmaPlot v11.0 software from Systat Software GmbH (Erkrath, Germany). Values of *p* < 0.05 were considered significant.

## 3. Results

### 3.1. HPTLC Analysis

#### 3.1.1. Method Development

A rapid and sensitive HPTLC method was developed to quantify the PDs present in RCE. The method was effective for the estimation of 20E, PA, and TU.

**Figure 2:**
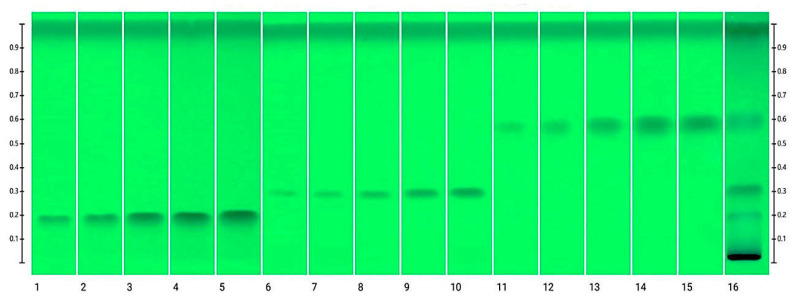
Comparison between different concentration levels of the standard solutions of PDs and RCE, where 1. TU 0.5 μg·band^−1^; 2. TU 0.75 μg·band^−1^; 3. TU 1 μg·band^−1^; 4. TU 1.25 μg·band^−1^; 5. TU 1.5 μg·band^−1^; 6. 20E 0.5 μg·band^−1^; 7. 20E 0.75 μg·band^−1^; 8. 20E 1 μg·band^−1^; 9. 20E 1.25 μg·band^−1^; 10. 20E 1.5 μg·band^−1^; 11. PA 0.5 μg.band^−1^; 12. PA 0.75 μg·band^−1^; 13. PA 1 μg·band^−1^; 14. PA 1.25 μg·band^−1^; 15. PA 1.5 μg·band^−1^; 16. RCE.

**Figure 3:**
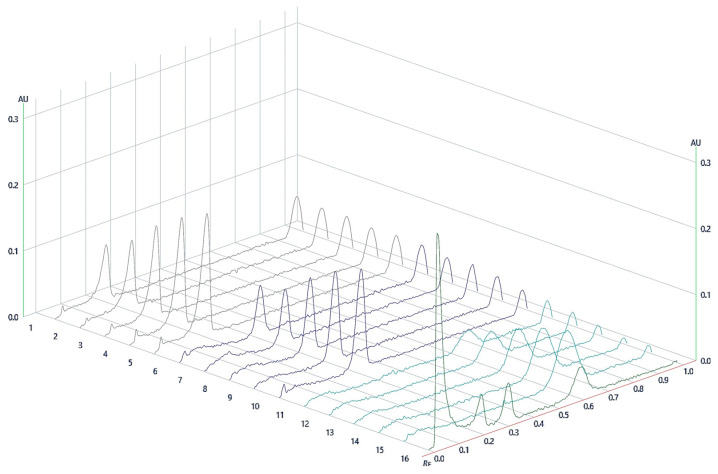
Profiles of different concentration levels of the standard solutions of PDs and RCE, where 1. TU 0.5 μg·band^−1^; 2. TU 0.75 μg·band^−1^; 3. TU 1 μg·band^−1^; 4. TU 1.25 μg·band^−1^; 5. TU 1.5 μg·band^−1^; 6. 20E 0.5 μg·band^−1^; 7. 20E 0.75 μg·band^−1^; 8. 20E 1 μg·band^−1^; 9. 20E 1.25 μg·band^−1^; 10. 20E 1.5 μg·band^−1^; 11. PA 0.5 μg·band^−1^; 12. PA 0.75 μg·band^−1^; 13. PA 1 μg·band^−1^; 14. PA 1.25 μg·band^−1^; 15. PA 1.5 μg·band^−1^; 16. RCE.

The first step of the method development was to choose a suitable solvent system for the analyzed compounds. For the determination of a suitable phase, various proportions of acetonitrile and methanol were used. Proportions including acetonitrile/methanol (85:15, *v*/*v*), acetonitrile/methanol (80:20, *v*/*v*), acetonitrile/methanol (10:90, *v*/*v*), acetonitrile/methanol (95:5, *v*/*v*), and acetonitrile/methanol (50:50, *v*/*v*) were investigated as the solvent systems for the development of a suitable band for quantitation. From the results, it was observed that the acetonitrile/methanol (85:15, *v*/*v*), acetonitrile/methanol (80:20, *v*/*v*), acetonitrile/methanol (10:90, *v*/*v*), acetonitrile/methanol (95:5, *v*/*v*), and acetonitrile/methanol (50:50, *v*/*v*) solvent systems presented a poor chromatogram of the examined PDs with a poor asymmetry factor. Among the tested solvent systems, acetonitrile/methanol (90:10, *v*/*v*) provided well-separated and compact chromatographic peaks of TU, 20E, and PA at Rf 0.2, 0.3, and 0.6, respectively. Hence, the acetonitrile/methanol (90:10, *v*/*v*) proportion was considered as a proper solvent system for the determination of 20E, PA, and TU in the HPTLC method. Figure 2 and Figure 3 represent the HPTLC chromatogram and profiles of different concentration levels of the PDs and RCE, respectively.

#### 3.1.2. Method Validation

The method was validated according to ICH guidelines [37].

##### Linearity

To establish linearity, an external standard curve was employed. The calibration curves were plotted by concentrations and the peak area of each PD. To prepare the standard solution, 20E (0.5 to 1.5 μg·band^−1^), PA (0.5 to 1.5 μg·band^−1^), and TU (0.5 to 1.5 μg·band^−1^) were dissolved in acetonitrile. The regression line was calculated with y = ax ± b, where x is the concentration and y is the peak area of each PD, b is the y-intercept, and a is the slope of the regression line. Moreover, the coefficient of determination (R^2^) was established for the linearity.

The linearity of the method was determined in the 0.5–1.5 μg.band^−1^ range for the tested substances. The regression line equation and the R^2^ for 20E were y = 0.0027x + 0.0002 and R^2^ = 0.9988, for PA, they were y = 0.0042x + 0.0003 and R^2^ = 0.9986, and for TU, they were y = 0.004x + 0.0008 and R^2^ = 0.997, respectively. These results showed a significant correlation and demonstrated the reliability of the method for estimating these PDs. Table 1 presents the results of the regression analysis, LD, and LQ.

##### Accuracy

Accuracy was established across the specified range of the analytical procedure, which was determined to be from the 0.5 to 1.5 μg·band^−1^ for 20E, PA, and TU.

The accuracy of the suggested HPTLC method was evaluated using the percentage of recovery of three concentration levels (low, medium, and high) with six replicates of each concentration. For the accuracy test, from each examined substance, three different quality control (QC) levels were used: lower QC (LQC: 0.75 μg·band^−1^), middle QC (MQC: 1 μg·band^−1^), and high QC (HQC: 1.25 μg·band^−1^) with six replicates. Table 2 presents the results of the accuracy of the developed HPTLC method.

##### Precision

The precision of the proposed HPTLC method was evaluated for both intra-day and inter-day precision, with six replicates of the injection. Examining the intra-day variation for the examined substances involved quantifying fresh solutions at LQC, MQC, and HQC on the same day in six replicates (*n* = 6). Inter-day variability for the examined substances was examined using the quantitation of freshly generated solutions at LQC, MQC, and HQC on three consecutive days in six replicates (*n* =6). Table 3 presents the results for the precision of these PDs.

##### Detection Limit (DL) and Quantitation Limit (QL)

The detection limit and quantification limit were expressed by the standard deviation of the slope (σ) and the slope of the calibration curve (S) using the following formulas: DL = 3.3 σ/S and QL = 10 σ/S, respectively.

The lowest concentrations for which a reliable spot was established were 0.11 μg·band^−1^ for 20E, 0.13 μg·band^−1^ for PA, and 0.04 μg·band^−1^ for TU. The quantification limit for 20E was 0.35 μg·band^−1^, for PA, it was 0.39 μg·band^−1^, and for TU, it was 0.12 μg·band^−1^, as shown in Table 1.

##### Robustness

The robustness of the proposed method was assessed by deliberately introducing variations in the mobile phase compositions and total run length. The solvents ratio of acetonitrile/methanol (90:10, *v*/*v*) was modified within a range of ±1%, and the HPTLC response was recorded for each set of conditions. The total solvent distance was altered to 72 mm and 68 mm from the initial 70 mm, and the HPTLC response was recorded. The observed changes in Rf values were within the range ± 0.02, which indicated that the method was robust.

In order to assess the stability of the standard solutions, they were stored at 2–8 °C for a week, visual inspection confirmed the clarity of the solutions, and subsequently, the obtained chromatograms from the freshly prepared solutions were compared with those derived from the stored solutions. The comparative analysis revealed that the samples maintained their stability throughout the entire duration of storage.

The close values of correlation factors to one, the high percentage of accuracy, and the low values of standard deviation suggested that the developed method is linear, accurate, precise, and reliable for the determination and quantification of 20E, PA, and TU. The developed and validated method was used for the quality determination of the three compounds in RCE. The amount of 20E was found to be 2.96 mg·g^−1^, PA was found to be 1.75 mg·g^−1^, and the amount of TU was found to be 1.65 mg·g^−1^ crude dry extract. The obtained HPTLC results were confirmed through HPLC/UV analysis using the previously validated method [42].

### 3.2. Effect of RCE, 20E, PA, and TU on Cell Viability

**Figure 4:**
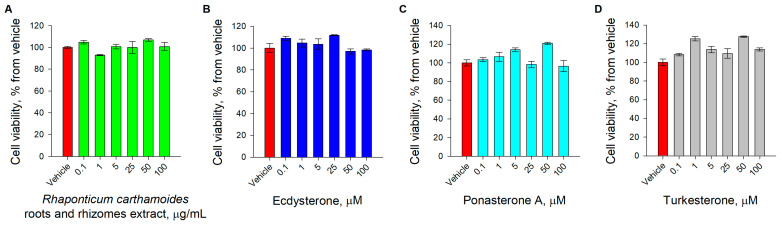
Cell viability was not affected upon RCE, 20E, PA, and TU treatment. Cell viability, expressed as the percentage of cell viability compared to the vehicle as the mean ± SEM upon treatment with (**A**) RCE, (**B**) 20E, (**C**) PA, and (**D**) TU.

The performed MTT assay revealed that the cell viability of the near-confluent preadipocytes was not affected upon incubation for 48 h with RCE μg·mL^−1^ and 20E, PA, and TU in 0.1–100 μM, respectively (Figure 4). Consequently, the selected treatment concentrations are safe for application in the following experiments.

### 3.3. Effect of RCE, 20E, PA, and TU on Adipogenesis in Human Adipocytes

**Figure 5:**
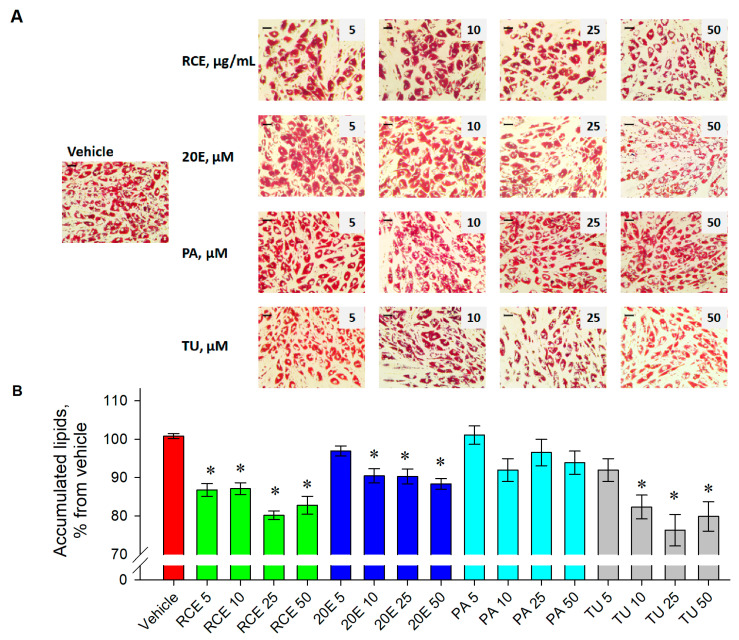
A significant decrease in adipogenic differentiation and lipid accumulation was observed upon RCE, 20E, and TU treatment. Representative microphotographs from the Oil red O staining of the experimental groups, 20x magnification (**A**). Quantification of accumulated lipids through spectrophotometric measures of the absorbance of the extracted Oil red O dye at 495 nm, represented as a percentage from vehicle (**B**). The data are expressed as the mean ± SEM. * *p* < 0.05 compared to the vehicle control group.

The chemical profiling of RCE affirmed the presence of 20E, PA, and TU. To evaluate whether RCE and the identified PDs modulate adipogenesis, ORO lipid staining was performed. The observed tendency toward a reduction in accumulated lipids is represented through microscopic images of the treated groups (Figure 5A).

The results of total lipid quantification (Figure 5B) revealed a statistically significant reduction upon the administration of the following treatments—RCE (86.8, 87.1, 80.2, and 82.8% for 5, 10, 25 and 50 μg·mL^−1^, respectively), 20E (97, 90.4, 90.2, and 88.3% for 5, 10, 25 and 50 μM, respectively), and TU (91.9, 82.3, 76, and 79.9% for 5, 10, 25 and 50 μM, respectively). Among the investigated treatments, the highest anti-adipogenic activity was observed for TU, followed by RCE and 20E. In the current experiment, PA did not affect adipogenic differentiation in human adipocytes.

Collectively, the screening, based on lipid staining, affirmed that RCE and the identified PDs—20E and TU—possess promising anti-adipogenic activity. Further experiments evaluated whether the modulation of adipolysis is involved in the observed decrease in total lipid content.

### 3.4. Effect of RCE, 20E, PA, and TU on Basal and Isoproterenol-Stimulated Lipolysis in Human Adipocytes

To determine the effect of RCE, 20E, PA, and TU on basal and isoproterenol-stimulated lipolysis, quantification of glycerol and FFAs released in the culture media was performed. 

**Figure 6:**
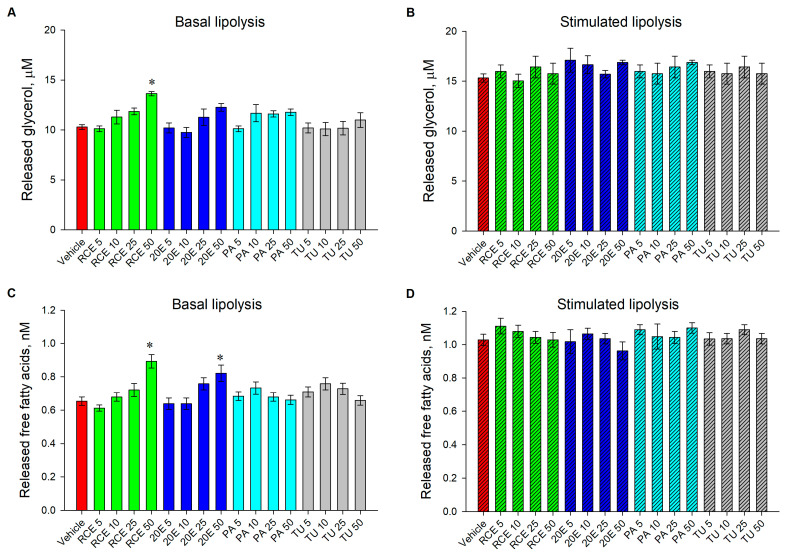
Only RCE and 20E stimulated basal lipolysis in human adipocytes. Glycerol concentration (µM) in cell culture media on day 9 of differentiation under basal conditions (**A**) and isoproterenol stimulation (10 µM for 24 h) (**B**). Free fatty acids concentration (nM) in cell culture media on day 9 of differentiation under basal conditions (**C**) and isoproterenol stimulation (**D**). The data are expressed as the mean ± SEM. * *p* < 0.05 compared to the vehicle control group.

Incubation with RCE (50 μg·mL^−1^) significantly increased the concentration of both glycerol (Figure 6A) and FFAs (Figure 6C) in the culture media under basal conditions. In a similar manner, in unstimulated adipocytes, 20E (50 μM) application only significantly increased the FFA concentration (Figure 6C). Treatment with PA and TU affected neither glycerol (Figure 6A) nor FFA (Figure 6C) concentrations. Therefore, we can suggest that both RCE and 20E significantly increased basal lipolysis, while such an effect was not observed upon PA or TU treatment (Figure 6A,C). Isoproterenol stimulation elevated the released glycerol and FFAs in comparison to the basal level. However, no significant effect on both glycerol (Figure 6B) and FFAs (Figure 6D) was detected upon all the treatments applied compared to the isoproterenol-stimulated vehicle.

Basal lipolysis was elevated only in the highest concentrations of RCE and 20E. However, none of the treatments affected isoproterenol-stimulated adipolysis.

## 4. Discussion

Considering the continuously increasing interest in plant products as food supplements due to the assumption of their safety [43], it is crucial to address the limited data regarding the control of their quality. Moreover, a lack of relevant scientific evidence confirming the biological activity and efficacy of some plant products [44] raises additional questions about the rationale of their use. Therefore, the development of fast, precise, and sensitive analytical methods is of great importance for the adequate control of plant products. RCE and PDs are widely used as adaptogens [22,45]. However, the available HPTLC methods for identifying and quantifying PDs, especially for PDs isolated from RCE, are currently quite limited, as evidenced by the data presented in Table 4 [46,47,48,49,50]. For that reason, a new HPTLC method that offers high sensitivity, efficiency, and reproducibility has been developed. The HPTLC technique has several advantages, including rapidity, cost-effectiveness, etc. [51,52], making it a highly suitable method for the future analysis of PDs. 

Except for the traditional adaptogenic activity of RCE, diverse biological activities are reported either for the extract or for the PDs investigated in this study. The extract has been evaluated for anti-neoplastic activity [53,54], cardioprotective effects [55], and the stimulation of muscle protein synthesis [56]. Moreover, an in vivo study indicates the beneficial effects of RCE on fat tissue expansion and hepatic triglyceride accumulation [57]. The reported biological activity of 20E includes anti-neoplastic activity [31,58], the modulation of mitochondrial bioenergetics [59], immunomodulatory effects [60], an increase in the muscle mass amelioration of the radiation-induced damage of oral mucosa [61], and neuroprotective [30,62,63], anti-fibrotic [64], wound-healing [65], and anti-inflammatory [66,67] activities. Several reports have proposed the potential of 20E to benefit metabolic disturbances such as obesity [36,68]; in addition, it has been reported to exert anti-diabetic [36,65,69,70] and anti-osteoporotic [71,72,73] effects. Moreover, as a food supplement in humans, 20E was found to increase strength performance with no effect on steroid profile [32]. Both PA and TU have not been investigated as natural compounds with anti-obesogenic effects.

The balance between adipogenesis and adipolysis determines the size of fat cells [74]. Thus, a decrease in lipid accumulation along with the stimulation of triglyceride mobilization are among the anti-obesogenic mechanisms of plant extracts and their constituents [75]. In order to affirm the available data for the potential of RCE and 20E in obesity management, as well as to evaluate the anti-adipogenic activity of PA and TU, the current investigation assessed the effect of RCE, 20E, PA, and TU on adipogenesis and lipolysis in vitro in human adipocytes. The applied cell-based platform provides fast and reliable screening of anti-adipogenic potential and accelerates the identification and selection of drugs which leads to their effects being subsequently evaluated in vivo.

The observed anti-adipogenic activity of RCE is in accordance with the previously reported decrease in the weight of epididymal fat tissue in rats [57]. Moreover, the obtained results suggested that among the identified PDs from the extract, only 20E and TU significantly decreased lipid accumulation during adipocyte differentiation. The detected effect upon 20E treatment is consistent with the literature data for reduced adipocyte size in diet-induced obesity in a murine model [68]. Interestingly, the current investigation suggested the notable anti-adipogenic activity of TU, which has not been previously reported.

Lipolysis is the process of triglyceride hydrolysis which is assumed to decrease the size of adipocytes [75] and can also be accepted as an indicator of energy expenditure [76]. Principally, there are two types of adipolysis—basal and stimulated (upon β3-adrenergic receptor activation by isoproterenol or catecholamines) [75]. Our findings suggest that RCE and 20E elevated basal lipolysis, which apparently contributes to the observed decrease in total lipid content.

In the current study, in comparison to 20E and TU, PA had no effect on adipogenesis and adipolysis, which have not been reported to our knowledge. Despite the common ecdysteroid structure in the investigated natural compounds, we could suggest that the observed difference in biological response is attributed to the lack of hydroxyl group on position 25 in PA, compared to 20E and TU.

The results of the present study demonstrated that RCE and 20E exhibit anti-obesity potential by reducing adipogenesis and promoting lipolysis in human adipocytes, while turkesterone promotes only adipogenesis. Further investigation is needed to fully understand the mechanism and affirm its potential therapeutic applications. Nevertheless, the findings highlight the importance of exploring the diversity of plant metabolites for drug discovery and development and suggest that *R. carthamoides* could be a promising source of natural anti-obesity agents or a combination of the most abundant secondary metabolites.

## 5. Conclusions

In summary, the developed and validated HPTLC method was demonstrated to be reliable for the estimation and quantification of the PDs 20E, PA, and TU. Using the developed HPTLC method, identification and quantification of these PDs in RCE were performed. Additionally, evaluation of anti-adipogenic activity revealed that RCE, 20E, and TU considerably decreased lipid accumulation in human adipocytes. Further experiments indicated that RCE and 20E significantly stimulated basal lipolysis, while no effect was observed upon PA and TU application. The obtained results from RCE, 20E, and TU are worth further mechanistic evaluation, which would provide a scientific rationale for subsequent in vivo experiments.

## Figures and Tables

**Table 1:** Linearity, LD, and LQ of the developed HPTLC method.

Parameter	20-Hydroxyecdysterone	Ponasterone A	Turkesterone
Range	0.5–1.5 μg·band^−1^	0.5–1.5 μg·band^−1^	0.5–1.5 μg·band^−1^
Regression line	y = 0.0027x + 0.0002	y = 0.0042x + 0.0003	y = 0.004x + 0.0008
R^2^	0.9988	0.9986	0.997
LD	0.11 μg·band^−1^	0.13 μg·band^−1^	0.04 μg·band^−1^
LQ	0.35 μg·band^−1^	0.39 μg·band^−1^	0.12 μg·band^−1^

**Table 2:** Evaluating the accuracy of the developed HPTLC method.

Concentration (μg·band^−1^)	Mean (μg.band^−1^) ± SD	Recovery %	CV%
20-hydroxyecdysone			
1.25	1.24 ± 0.010	99.06	0.82
1	0.99 ± 0.008	99.07	0.84
0.75	0.76 ± 0.008	100.90	1.10
Ponasterone A			
1.25	1.25 ± 0.006	99.62	0.49
1	0.99 ± 0.009	98.65	0.89
0.75	0.76 ± 0.007	100.95	0.93
Turkesterone			
1.25	1.26 ± 0.014	100.97	1.12
1	0.99 ± 0.010	99.21	1.01
0.75	0.74 ± 0.012	98.83	1.70

CV%—percent of coefficient of variation.

**Table 3:** The precision of the developed HPTLC method.

Concentration (μg·band^−1^)	Intraday Precision			Interday Precision		
	Mean (μg·band^−1^) ± SD	SE	CV%	Mean (μg·band^−1^) ± SD	SE	CV%
20-hydroxyecdysone						
1.25	1.24 ± 0.011	0.004	0.86	1.24 ± 0.011	0.003	0.95
1	1.00 ± 0.009	0.004	0.86	0.99 ± 0.009	0.004	0.96
0.75	0.75 ± 0.008	0.003	1.02	0.75 ± 0.010	0.004	1.27
Ponasterone A						
1.25	1.25 ± 0.008	0.003	0.62	1.25 ± 0.007	0.003	0.53
1	0.99 ± 0.008	0.003	0.85	0.99 ± 0.008	0.003	0.77
0.75	0.75 ± 0.010	0.004	1.37	0.75 ± 0.009	0.004	1.16
Turkesterone						
1.25	1.25 ± 0.010	0.004	0.79	1.24 ± 0.010	0.004	0.82
1	0.99 ± 0.017	0.007	1.66	0.99 ± 0.013	0.005	1.31
0.75	0.74 ± 0.008	0.003	1.06	0.74 ± 0.010	0.004	1.29

CV%—percent of coefficient of variation; SE—standard error.

**Table 4:** Comparison between chromatographic conditions of the HPTLC methods for PD determination.

Purpose	Chromatographic Conditions	LD/LQ	Ref.
Ecdysteroids (20E, ponasterone A, and others) characterization of some *Silane* species	RP-HPTLC plates, mobile phase: chloroform: ethanol 4:1 (*v*/*v*), visualized under 254 nm.	*-*	[46]
Ecdysteroids (20E, ponasterone A, turkesterone, and others) characterization of some *Silane* species	RP-HPTLC plates, ethanol: water 3:2 (*v*/*v*) and acetone: water 3:2 (*v*/*v*), visualized under 254 nm.	-	[47]
Determination and quantitation of 20E in *Sida rhombifolia* L. and dietary supplements	HPTLC plates were prewashed with methanol and dried in an oven at 120 °C for three minutes, mobile phase: chloroform: methanol 8:2 (*v*/*v*), distance 60 mm, visualized under 250 nm.	LD 60 ng·spot^−1^ LQ 200 ng·spot^−1^	[48]
Development and validation of an HPTLC method for the quantification of 20E	HPTLC plates, mobile phase: THF: toluene: 1 mM TFA in methanol: water 16:8:2:1 (*v*/*v*/*v*/*v*), a distance of 70 mm, visualized under 250 nm.	Lower limits of quantitation—70–100 μg·mL^−1^ Upper limits of quantitation—815 μg·mL^−1^ above 1000 μg·mL^−1^.	[49]
Monitoring of ecdysteroids isolated from *Manduca sexta* pupae	HPTLC plates, mobile phase: chloroform: ethanol (65:35, *v*/*v*), chloroform: methanol: 10-N-ammonium hydroxide 28:20:2 (*v*/*v*/*v*) for ecdysteroid acids and 15:35:3.5 (*v*/*v*/*v*) for ecdysteroid conjugates, visualized under UV light and sprayed with 50% sulfuric acid solution.	-	[50]

## Data Availability

Not applicable.
